# Dual Tracers of 16α-[18F]fluoro-17β-Estradiol and [18F]fluorodeoxyglucose for Prediction of Progression-Free Survival After Fulvestrant Therapy in Patients With HR+/HER2- Metastatic Breast Cancer

**DOI:** 10.3389/fonc.2020.580277

**Published:** 2020-10-29

**Authors:** Cheng Liu, Xiaoping Xu, Huiyu Yuan, Yongping Zhang, Yingjian Zhang, Shaoli Song, Zhongyi Yang

**Affiliations:** ^1^Department of Nuclear Medicine, Fudan University Shanghai Cancer Center, Shanghai, China; ^2^Department of Oncology, Shanghai Medical College, Fudan University, Shanghai, China; ^3^Center for Biomedical Imaging, Fudan University, Shanghai, China; ^4^Shanghai Engineering Research Center of Molecular Imaging Probes, Shanghai, China; ^5^Department of Nuclear Medicine, Shanghai Proton and Heavy Ion Center, Shanghai, China

**Keywords:** heterogeneity, ER expression, breast cancer, FES/FDG, fulvestrant

## Abstract

**Objective:**

The purpose of this study was to employ dual tracers 16α-[18F]fluoro-17β-estradiol (^18^F-FES) and [18F]fluorodeoxyglucose (^18^F-FDG) as imaging biomarkers in predicting progression-free survival (PFS) in ER-positive metastatic breast cancer (MBC) patients receiving fulvestrant therapy.

**Methods:**

We retrospectively analyzed 35 HR+HER2- MBC patients who underwent ^18^F-FES and ^18^F-FDG PET/CT scans prior to fulvestrant therapy in our center. The SUVmax across all metastatic lesions on the PET/CT were assessed. The heterogeneity of ER expression was assigned by the presence of any ^18^F-FES negative lesions for patients with entirely ^18^F-FES positive lesions categorized into two groups by the median ratio of FES/FDG SUVmax, low FES/FDG, and high FES/FDG. PFS were estimated by the Kaplan-Meier method and compared by the log-rank test. Univariate and multivariate analyses were performed using the Cox proportional hazard model.

**Results:**

In total, 12 patients had both ^18^F-FES negative and positive lesions, indicating the heterogeneity of ER expression in metastatic lesions. These patients had a low median PFS of 5.5 months (95% CI 2.3–8.7). Of patients with entirely ^18^F-FES positive lesions, 11 had a low FES/FDG, and 12 had a high FES/FDG. These groups had a median PFS of 29.4 months (95% CI 2.3–56.5) and 14.7 months (95% CI 10.9–18.5), respectively. The patients were stratified in three categories based on incorporating both ^18^F-FES and ^18^F-FDG imaging results that were significantly correlated with PFS by univariate analysis (*P* < 0.001) and multivariate analysis (*P* = 0.006).

**Conclusion:**

^18^F-FES and ^18^F-FDG PET could serve as prognostic imaging biomarkers for ER-positive MBC patients treated with fulvestrant therapy.

## Introduction

Breast cancer is the most common cancer in women worldwide. According to U.S. cancer statistics, about 276,480 newly diagnosed cases are estimated in 2020, resulting in approximately 42,170 deaths (1). It is the second most common cause of cancer death in women. Approximately 70%–80% of breast cancers are hormone receptor (HR)-positive, and endocrine therapy plays a vital role in the management of such cancers (2).

Fulvestrant, a pure anti-estrogen drug that exerts no partial agonist effects, is approved for postmenopausal women with HR+ metastatic breast cancer (MBC) and disease progression following the prior failure of other endocrine therapy (3, 4). Although many patients have a prolonged clinical response to fulvestrant, there are still some patients who are unable to benefit or develop resistance. Therefore, the identification of clinical or molecular markers that predict which patients with MBC might benefit from Fulvestrant is vitally important because it helps to individualize treatment and could significantly improve the management of breast cancer. The level of ER expression has been shown to provide important prognostic information, and in most cases, higher levels of tumor ER expression are associated with more noteworthy clinical benefit from conventional endocrine therapy (5). A biopsy was routinely utilized to discriminate between ER-positive and ER-negative lesions. However, this gold standard method may not be representative of ER heterogeneity. Furthermore, collecting a biopsy sample from metastatic tissue is not always feasible in daily practice because of the characteristics of lesion location and the risk associated with biopsy.

Positron emission tomography (PET) with 16α-[18F]fluoro-17β-oestradiol (^18^F-FES) has been proposed as a noninvasive method to visualize and quantify ER expression in recurrent or metastatic lesions (6, 7). Early clinical studies focused on sole ^18^F-FES PET imaging to predict clinical response to endocrine therapy, rarely performed in combination with ^18^F-FDG imaging (8–10). Kurland and colleagues evaluated the ability of ^18^F-FDG and ^18^F-FES to predict progression-free survival (PFS) in 84 patients treated by salvage endocrine therapy for ER-positive MBC (11). They summarize that, although ^18^F-FES PET is not predictive of the patient’s PFS in the whole population, it is meaningful that this imaging could stratify the patients with high FDG uptake. However, this study fails to discuss the response to different endocrine therapies because of differences in the pharmacodynamics of different ER antagonists (12). We have previously reported that early change in SUVmax of ^18^F-FES PET/CT could be used to predict response to fulvestrant (13). Nevertheless, this method still requires a 28-day period of fulvestrant treatment before the effect can be observed. Therefore, the purpose of our study was to evaluate the clinical value of dual tracers ^18^F-FDG and ^18^F-FES at baseline in predicting the response of fulvestrant in HR-positive MBC patients.

## Methods

### Patients

In this retrospective analysis, we evaluated 35 HR+/HER2- MBC patients who were treated with 500 mg fulvestrant and underwent both ^18^F-FES PET/CT and ^18^F-FDG PET/CT scans within 4 weeks before initiating treatment between May 2016 and March 2019. The lag time between the two scans was within 1 week. All data were retrospectively collected from the medical records. To ensure the sensitivity and specificity of ^18^F-FDG and ^18^F-FES imaging, patients with ER antagonist discontinuation for less than 5 weeks and medical comorbidities (diabetes, a chronic infection, or chronic inflammatory conditions) were not enrolled in this study (12, 14). The enrolled patients had performed ^18^F-FES scans for one of the following purposes: 1) predicting response to fulvestrant, a phase II study (NCT03507088, *n* = 23) or 2) identifying the ER status of metastatic lesions for clinical practice (*n* = 12). The study was approved by the Fudan University Shanghai Cancer Center Ethic Committee and Institutional Review Boards for clinical investigation, and the need for informed consent was waived as it is a retrospective study.

### Fulvestrant and Clinical Follow-Up

Fulvestrant 500 mg was administered by intramuscular injection on days 1, 15, and 29 and every 28 days after that. For premenopausal women, patients received concurrent luteinising hormone-releasing hormone analogues (LHRHa). Treatment continued until progressive disease (PD) or other criteria for discontinuation were met in terms of adverse events or a patient’s decision to withdraw.

Clinical follow-up was performed every 3 months by radiologic imaging (e.g., diagnostic CT, MRI, bone scan, ^18^F-FDG), serum tumor markers, and evaluation of symptoms until disease progression or death. PFS was defined as the time from fulvestrant treatment to disease progression or death from any cause. For patients with measurable disease, tumor response was determined by an experienced radiologist according to the Response Evaluation Criteria in Solid Tumors (RECIST) version 1.1 and was blinded to the results of baseline ^18^F FES and ^18^F FDG PET/CT. Patients with only non-measurable lesions were considered to have disease progression when there was a definite progression of existing lesions or when new lesions were detected at follow-up.

### PET/CT Procedure

The synthesis and quality control of ^18^F-FDG and ^18^F-FES were performed as reported in our previous study (15).

^18^F-FDG PET/CT imaging was done according to standard clinical procedures. All patients fasted for at least 6 h and had serum glucose levels less than 10 mmol/L before the intravenous injection of ^18^F-FDG (3.7–7.4 MBq/kg). The patients were kept lying comfortably in a quiet, dimly lit room before and after the tracer injection. About 1 h after tracer injection, the patients were administered 1 L of plain water orally and then scanned in the PET/CT (Siemens Biograph 16HR PET/CT or mCT Flow PET/CT scanner). About 222 MBq of ^18^F-FES was injected intravenously over 1–2 min. The scanning was initiated 1 h after administration of the tracer on the same PET/CT scanner as the ^18^F-FDG. The detail of PET/CT acquisition parameters were described as reported in prior studies (16).

### Image Analysis

PET images were reviewed and analyzed by two board-certified nuclear medicine physicians using a multimodality computer platform (Syngo, Siemens, Knoxville, TN, USA). All parameters were assessed in 3-dimensional volumes. Regions of interest (ROI) were manually drawn over lesions by an experienced nuclear medicine physician using the PET images with the corresponding noncontrast CT serving as a guide, and the contours of lesions were checked for concurrence by a second experienced nuclear medicine physician. In case of a discrepancy between the two physicians, consensus was reached on a final reading for the statistical analyses. Semiquantitative analysis of tumor metabolic activity was obtained using standardized uptake value (SUV) normalized to body weight. A lesion showing uptake intensity higher than with adjacent normal tissue background was defined as positive for ^18^F-FDG and ^18^F-FES, and hypermetabolic foci estimated by inflammatory or physiologic activity were not considered. We used the cutoff value of SUVmax ≥ 1.8 to define ^18^F-FES positivity and quantify the ER expression based on our previous study (17). Lesions seen on ^18^F-FES and ^18^F-FDG PET/CT images were also identified and localized by other conventional imaging techniques (bone scan, diagnostic CT, MRI, or ultrasound). In patients with extensive metastatic lesions, an arbitrary maximum of 20 randomly chosen lesions of ^18^F-FDG PET correspond to the ^18^F-FES avid lesions according to the guidelines of the European Association of Nuclear Medicine (EANM) (18). Due to high physiological ^18^F-FES uptake, patients with liver lesions were not included in the ^18^F-FES analysis (19).

### Statistical Analysis

All PET imaging parameters were dichotomized using the median as a threshold. For patients with entirely FES positive lesions, the FES/FDG ratio of each tumor was calculated, and the median value was selected as the cutoff to distinguish between high and low FES/FDG.

The survival analyses were estimated by the Kaplan–Meier method and compared by the log-rank test (image parameters and demographic factors). Univariate and multivariate analyses were estimated using the COX proportional hazards model and expressed as a hazard ratio with corresponding 95% confidence intervals and *P* values. Multivariate analysis with the stepwise model by forward selection was performed with those variables that had proven significant on univariate analysis to explore independent predictors of PFS. All data analyses were performed using IBM SPSS Statistics software, version 20.0 (IBM Corporation, Armonk, NY, USA). Two-sided *P* values of less than 0.05 were considered to indicate statistically significant differences.

## Results

### Patient Characteristics and Treatment Outcome

The characteristics of the 35 enrolled MBC patients are listed in **Table 1**. At the time of analysis (Jan. 2020), 26 patients (74.3%) experienced progression, and all of them were radiologic PD. The median follow-up period was 9.5 months (range: 2.1–30.0), and the median PFS was 12.2 months (95% CI: 4.7–19.7). Twenty-six patients had measurable lesions according to RECIST version 1.1, four patients had non-measurable visceral lesions, and five patients had only bone metastases. Twenty-four of the 35 patients (68.6%) experienced clinical benefit from fulvestrant treatment as indicated by PFS ≥ 24 weeks. Furthermore, fulvestrant was well tolerated in all patients and no patients who discontinued treatment due to adverse events.

**Table 1:** Patient demographics and disease characteristics.

Characteristics	*N* = 35	%
**Median age, years**	56.0 (40-78)	
**Menopausal status**		
Premenopause^a^	7	20.0
Postmenopause	28	80.0
**Histology of primary breast cancer**		
Ductal	29	82.8
Lobular	4	11.4
Mucinous	1	2.9
Tubular	1	2.9
**DFI**^b^		
≤5 y	10	28.6
>5 y	16	45.7
**PgR status**		
Positive	31	88.6
Negative	4	11.4
**Metastatic sites**		
Non-visceral	25	71.4
Bone	20	57.1
Bone-only	5	14.3
Visceral disease	10	28.6
Any lung	7	20.0
Pleural	5	14.3
Liver	1	2.9
**No. of disease sites**		
1	16	45.7
2	13	27.1
≥ 3	6	17.1
**De novo metastatic disease**	9	25.7
**Prior line of therapies for metastatic disease**		
0	28	80.0
1	5	14.3
2	2	5.7
**Prior ET for metastatic disease**		
None	30	85.7
Yes	5	14.3
**Prior chemotherapy for metastatic disease**		
None	31	88.6
Yes	4	11.4
**PFS**		
Events	26	74.3
Censored	9	25.7

ER, estrogen receptor; PgR, progesterone receptor; ET, endocrine therapy; PFS, Progression-Free Survival; DFI, Disease-free interval.

^a^For premenopausal women, fulvestrant was given upon on the administration of gonadotropin-releasing hormone agonist.

^b^Patients with stage IV breast cancer at initial diagnosis were excluded (N = 9).

### PET/CT Analysis

In total, 235 metastatic lesions were identified in 35 patients. The number of lesions found per patient ranged from 1 to 20 with a median of 6 lesions per patient. Lesions were located in lymph nodes (*n* = 78), bones (*n* = 117), lungs (*n* = 15), pleural (*n* = 9), soft tissue (*n* = 15), and the liver (*n* = 1). All these metastatic lesions were ^18^F-FDG avid. In addition, using a cutoff value of SUVmax ≥ 1.82 to define ^18^F-FES positivity, 17 lesions were ^18^F-FES negative (nine lymph nodes, six bone lesions, one lung metastatic, and one soft tissue) in 12 (34.3%) of 35 patients, showing remarkable heterogeneity of ER expression in these metastatic breast cancer patients. Interestingly, one patient had liver metastases and also had FES-negative metastases elsewhere, so this patient was included in the 12 patients with heterogeneous ER expression.

On the ^18^F-FDG scan, the median SUVmax values among all lesions were 4.92 (range 1.68–40.74). On the ^18^F-FES scan, the median SUVmax values among 217 ^18^F-FES positive lesions (excluding 17 ^18^F-FES negative lesions and one liver metastatic) was 4.7 (range 1.8–22.8). On a per-patient level, the median SUVmax of ^18^F-FDG and ^18^F-FES was 4.4 (range 2.1–15.5) and 4.5 (range 2.0–13.5), respectively. For patients with entirely ^18^F-FES positive lesions, the median ratio of FES/FDG SUVmax was 0.96 (range 0.2–3.2). The detailed PET parameters of each patient are shown in **Supplemental Table 1**.

### Prediction of Response to Fulvestrant

We first examined the significance of conventional clinical parameters. Patients with disease-free interval (DFI) ≥ 5 years had a longer PFS compared to those with less time of DFI (median PFS 12.2 months vs. 3.1 months, *P* = 0.047). However, this was of borderline significance in univariate analysis (*P* = 0.054). Other clinical risk factors (age, menopausal status, presence of visceral disease, *de novo* metastatic disease, histology of primary breast cancer, number of disease sites, bone-only disease, prior palliative chemotherapy, and lines of endocrine therapy for MBC) were not significantly related to PFS (**Table 2**).

**Table 2:** Univariate and multivariate Cox regression analyses for prediction of progression-free survival (PFS).

Parameters	No.	Event	Median PFS	Log-rank	Univariate analysis		Multivariate analysis	
			(95% CI)	*P* value	HR (95% CI)	*P* value	HR (95% CI)	*P* value
**Age**								
<65	25	19	7.0(5.9-8.2)	0.318	0.64(0.27-1.54)	0.322	NA	
≥65	10	6	15.5(11.0-20.0)					
**Menopausal status**								
Pre-menopause	7	6	5.6(5.3-5.9)	0.416	0.68(0.27-1.73)	0.419	NA
Post-menopause	28	20	13.1(3.8-22.4)				
**Disease-free interval**								
≤5 y	10	10	3.1(0.0-6.4)	0.047*	0.42(0.17-1.01)	0.054	/	0.052
>5 y	16	11	12.2(1.5-22.9)					
**Histology of primary breast cancer**								
Ductal	29	22	9.5(0.1-18.9)	0.593	0.72(0.21-2.43)	0.595	NA	
Lobular	4	3	14.7(4.0-20.4)					
**No. of disease sites**								
1	16	11	12.2(2.7-21.6)	0.202				
2	13	9	13.1(3.7-22.5)		1.13(0.46-2.78)	0.267	NA	
≥3	6	6	5.6(4.7-19.7)		2.42(0.86-6.82)			
**Visceral disease**								
No	25	19	9.5(1.6-17.5)	0.440	0.71(0.29-1.71)	0.443	NA	
Yes	10	7	13.8(0.0-27.7)					
**Bone only disease**								
No	30	23	12.2(3.8-20.7)	0.709	1.26(0.24-2.68)	0.710	NA	
Yes	5	3	2.4(2.0-2.8)					
**De novo metastatic disease**								
No	26	21	7.0(0.0-14.3)	0.167	0.51(0.19-1.36)	0.176	NA	
Yes	9	5	18.4(10.9-25.9)					
**Prior palliative chemotherapy**								
No	31	22	12.2(3.4-21.0)	0.516	1.43(0.48-4.22)	0.518	NA	
Yes	4	4	6.6(0.0-16.8)					
**Lines of endocrine therapy for MBC**								
1	28	20	12.2(3.1-21.3)	0.479	1.39(0.55-3.51)	0.482	NA	
≥2	7	6	6.6(5.3-7.9)					
**FDG SUVmax**								
<4.4	17	12	15.5(9.2-21.8)	0.186	1.72(0.76-3.91)	0.192	NA	
≥4.4	18	14	6.6(5.6-7.6)					
**FES SUVmax**								
<4.5	17	13	12.2(2.9-21.5)	0.995	0.99(0.45-2.19)	0.995	NA	
≥4.5	18	13	7.0(0.0-20.5)					
**FES/FDG ratio**								
With FES negative	12	12	5.5(2.3-8.7)	<0.001*		<0.001*		0.006*
<0.96	11	6	29.4 (2.3-56.5)		0.09(0.03-0.32)		0.10(0.02-0.49) 0.27(0.09-0.78)	
≥0.96	12	8	14.7 (10.9-18.5)		0.22(0.08-0.59)			

PFS, progression-free survival; CI, confidence interval; HR, hazard ratio; MBC, metastatic breast cancer; SUVmax, maximum standard uptake value.

*P ≤ 0.05; N/A: Analysis not performed as univariate analysis not significant.

Next, we tested whether the PET parameters correlate with survival in patients treated with Fulvestrant. The cutoff value of SUVmax of determined by the median value of ^18^F-FDG and ^18^F-FES was 4.4 and 4.5, respectively. It is regrettable that neither of the single parameters of the two scans was significantly associated with PFS (*P* > 0.05) (**Table 2**). We also analyzed the data stratified by low/high FDG and FES SUVmax, and there is no predictive value of PFS (**Supplemental Figure 1**).

**Figure 1:**
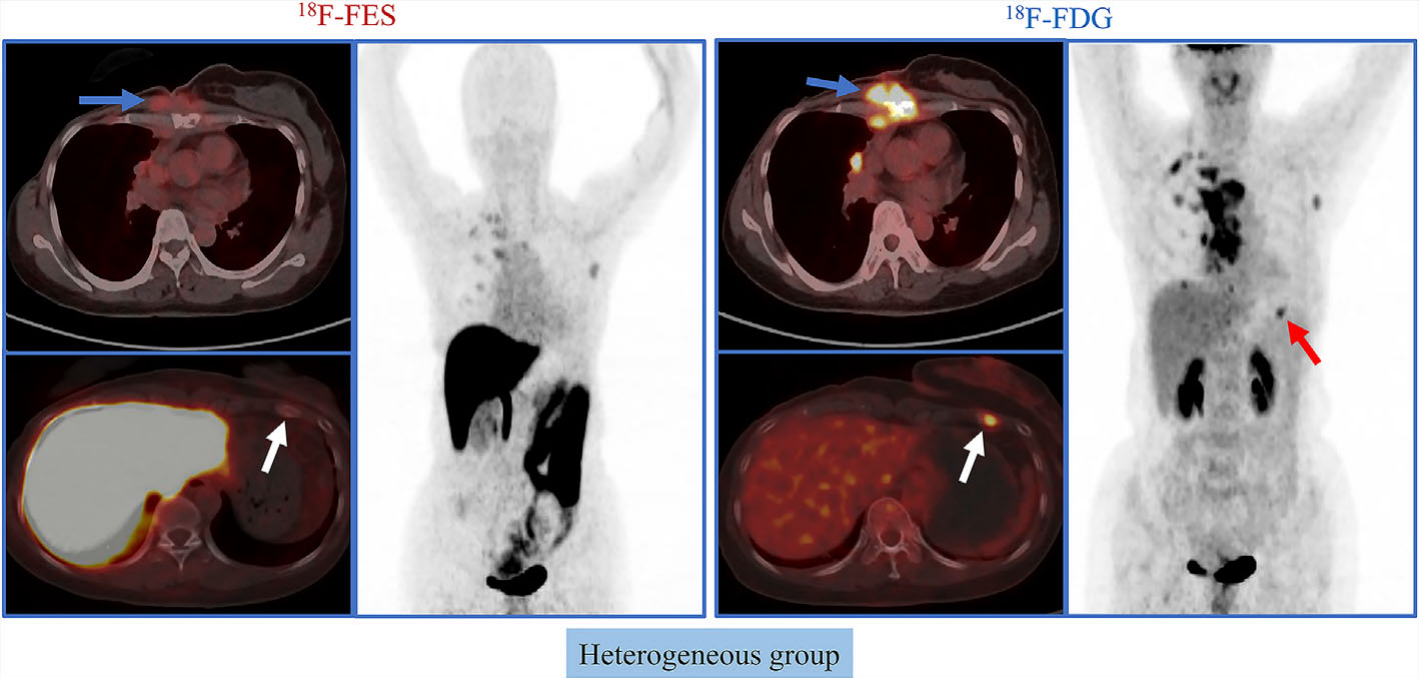
Representative cases of heterogeneous group. A 50-year-old female patient has both ^18^F-FES positive and negative lesions. The left rib shows significant uptake on FDG but not on FES. For this patient, the PFS was 3.7 months, and she did not receive clinical benefit from fulvestrant treatment.

**Figure 2:**
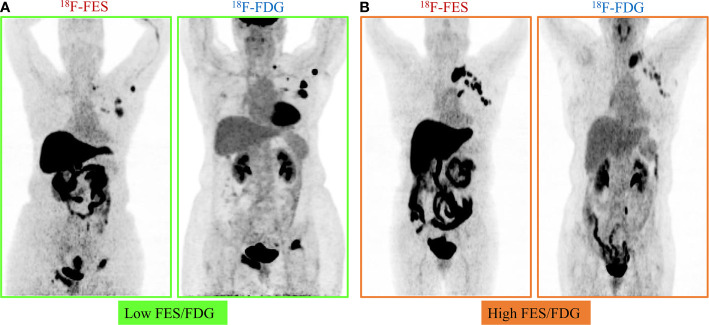
Representative cases of FES/FDG group. Patients with 100% of the ^18^F-FES positive metastatic lesions were divided into two groups by the median ratio of FES/FDG SUVmax (0.96). **(A)** Low FES/FDG. A 59-year-old female patient with the range of ^18^F-FDG and ^18^F-FES SUVmax was 5.3–40.7 and 4.1–15.5, respectively. This patient’s median FES/FDG was 0.52, which was lower than the median FES/FDG of all patients. She has received fulvestrant treatment for 27.6 months until progress. **(B)** High FES/FDG. A 67-year-old female patient with the range of ^18^F-FDG and ^18^F-FES SUVmax was 3.0–8.1 and 8.8–16.0, respectively. This patient’s median FES/FDG was 2.32, which was higher than the median FES/FDG of all patients, and the PFS was 14.7 months.

**Figure 3:**
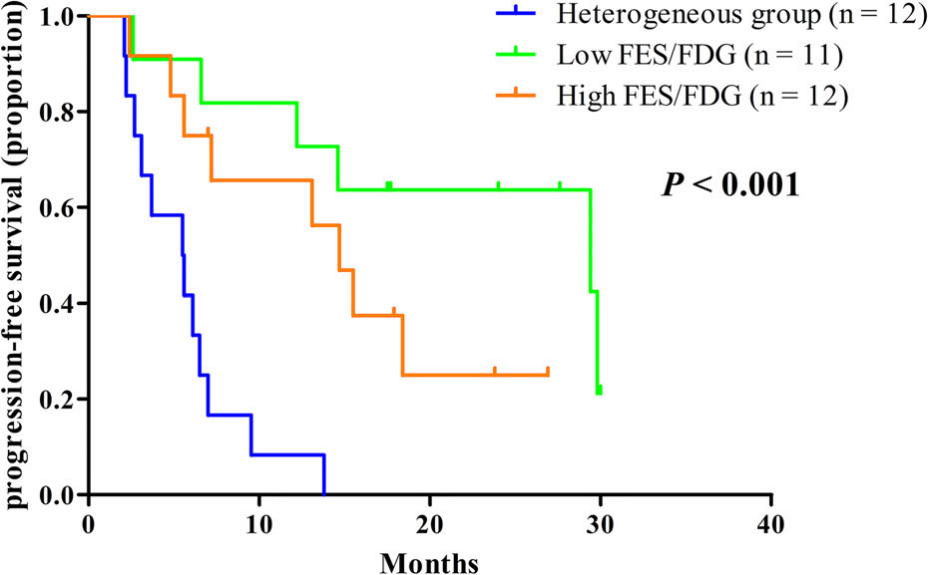
Kaplan-Meier curves of PFS stratified by the three classification groups. Heterogeneous group (*n* = 12, median PFS 5.5 months), low ratio of FES/FDG (*n* = 11, median PFS 29.4 months), high ratio of FES/FDG (*n* = 12, median PFS 14.7 months).

Given the significant heterogeneity of ER expression in these patients with metastatic breast cancer, they may fail to respond to endocrine therapy. The population was stratified in three categories: 1) The heterogeneous group (*n* = 12) had both ^18^F-FES negative and positive sites (**Figure 1**); 23 patients with entirely ^18^F-FES positive lesions further divided into two groups by the median FES/FDG SUVmax ratio (the median value was 0.96). 2) The other groups are the low FES/FDG group (FES/FDG < 0.96, *n*= 11, **Figure 2A**) and 3) the high FES/FDG group (FES/FDG ≥ 0.96, *n* = 12, **Figure 2B**). Patients with the heterogeneity of ER expression were significantly associated with shorter PFS compared to those without ^18^F-FES negative lesions in univariate analysis (*P* < 0.001, **Figure 3**). Median PFS was 5.5 months (95% CI 2.3–8.7) for the heterogeneous group, 29.4 months (95% CI 2.3–56.5) for the low FES/FDG group, and 14.7 months (95% CI 10.918.5) for the High FES/FDG (**Table 2**).

In multivariate analysis, a three-way PET classifier (FES heterogeneous, low FES/FDG, and high FES/FDG groups) remained the only independent, statistically significant prognostic factor for PFS (*P* = 0.006). Although DFI was a trend in the log-rank test, they were not considered as statistically independent prognostic factors (*P* = 0.052).

## Discussion

Our results have demonstrate that an integrated parameter derived from ^18^F-FDG and ^18^F-FES PET may have prognostic value for fulvestrant therapy in patients with ER-positive metastatic breast cancer. In our relatively small cohort, all clinical risk factors and single PET parameters were not significantly associated with PFS on multivariate analyses, whereas the PET classifier of ^18^F-FDG and ^18^F-FES remained significant.

Other scholars and our previous studies have confirmed that ^18^F-FES PET can noninvasively and systematically assess ER status in patients with recurrent or metastatic breast cancer, and as an imaging biomarker for predicting response to endocrine therapy (13, 17, 20, 21). Nevertheless, ^18^F-FES PET is challenging to monitor nonfunctional ER lesions, which might potentially lead to losing sight of ER-negative lesions. Some studies have used ^18^F-FDG PET/CT together with ^18^F FES-PET for the identification of ^18^F FES negative lesions (22).

To our knowledge, this is the first dual-tracer PET study evaluating the effect of fulvestrant on ^18^F-FDG and ^18^F-FES in patients with ER-positive MBC. Several previous studies have described the prognostic value of single ^18^F-FDG or ^18^F-FES PET in ER-positive MBC (9, 12, 13, 23). However, these studies have certain limitations, such as under a specific population or needng a period of treatment to play a predictive role. Another study investigates the utility of ^18^F-FDG and ^18^F-FES PET on variety endocrine therapy in patients with ER-positive MBC but did not attempt to predict the efficacy of fulvestrant precisely (11). Consistent with other ^18^F-FES PET studies, our results indicated that baseline ^18^F-FES SUVmax was not correlated with treatment outcome (9). The predictive value of ^18^F-FDG PET in patients with ER-positive MBC for fulvestrant therapy was proved by our previous study (23). In the current study, however, we did not find that sole ^18^F-FDG SUVmax provides independent prognostic information for fulvestrant. An explanation for this could be that the populations of the two studies were different because patients with only bone metastasis were excluded from the previous research.

Kurland and colleagues’ study demonstrated that the FES/FDG ratio appears to provide a reasonable summary of synchronous ER expression for patients with highly discordant ^18^F-FES uptake across tumor sites in predicting clinical response to endocrine therapy (22). Furthermore, based on our previous study it was proposed that the ratio of SUVmax-FES/FDG showing potential in predicting neoadjuvant chemotherapy response of breast cancer (24). Besides, one study has also suggested that patients with low or absent ^18^F-FES uptake in metastases may be unlikely to benefit from endocrine therapy (25). Our recent research showed that patients with entirely ^18^F-FES positive lesions have a median PFS that is nearly twice of patients with negative ^18^F-FES (14.6 months vs. 7.2 months), and the difference was of borderline significance (*P* = .081) (13). Hence, we hypothesized that the ratio of FES/FDG and the heterogeneous uptake of FES would predict response to fulvestrant therapy. Therefore, the current study combines the above two concepts into one classification scheme by sorting patients into three groups (heterogeneous disease, low FES/FDG, and high FES/FDG) based on ^18^F-FDG and ^18^F-FES PET scans. Our results suggest that, for ER-positive MBC, patients with the heterogeneity of ER expression by ^18^F-FES PET were unlikely to benefit from fulvestrant, and it may indicate that potential changes in ER expression of tumor in explaining endocrine therapy resistance, whereas patients with totally ^18^F-FES positive in metastases are potential candidates for fulvestrant, particularly those with low FES/FDG.

Our study reports an incremental refinement of the classifier by integrating both ^18^F-FES and ^18^F-FDG imaging results based upon a smaller cohort but a more uniformly treated patient population compared with previous studies. Kurland et al. demonstrate that patients with low FDG uptake (indolent tumors) had a longer median PFS with high FDG uptake and high average FES uptake had a moderate median PFS and with high FDG uptake and low average FES uptake had a shorter median PFS (11). Nevertheless, this study differed from our current research in several respects. Patients had received different kinds of endocrine therapy, including aromatase inhibitor combined with or without fulvestrant, tamoxifen, and fulvestrant. Moreover, patients with the heterogeneity of ER expression were not individually analyzed, and those patients tended to develop resistance to endocrine therapy. We, therefore, analyzed those patients with the heterogeneity of ER expression independently, which may not benefit from sole fulvestrant, and it might be better to change management by adding complementary treatments, such as chemotherapy, everolimus, or cyclin-dependent kinases 4 and 6 (CDK4/6) inhibitors. In the current study, we report that patients with 100% ^18^F-FES positive and low FES/FDG had a longer median PFS (29.4 months, 95% CI 2.3–56.5) compared with high FES/FDG (14.7 months, 95% CI 10.9–18.5). Consistent with our previous study, patients with high baseline ^18^F-FDG tumor uptake had a longer PFS (23); one of the possible reasons is that 17β-estradiol (E2) increases ER-dependent PI3K/Akt activation-mediated Glucose uptake signaling pathway in HR-positive breast cancer cell lines (26).

Increasing evidence suggests that, in addition to ER expression, progesterone receptor (PR) expression may also be related to the prognosis of fulvestrant therapy (27). The Zhao et al. study had reported that the ^18^F-FDG/^18^F-FES SUV ratio was correlated with ERα, PR expression (28). Therefore, the FES/FDG ratio may be more representative of comprehensive ER, PR expression, and could be a potential imaging biomarker to predict survival on fulvestrant therapy in patients with HR-positive breast cancer.

There were several limitations to our study. First, the retrospective nature of this study and the heterogeneous patient population, perhaps with inherently different prognostic factors, are a major limitation. Our study shows that patients with a high FES/FDG ratio have shorter survival than those with a low FES/FDG ratio. This is the opposite of what we expected: that patients with greater FES-avidity and lower FDG-avidity would be expected to lead to longer survival. However, most of the current studies indicate that patients with FES negative or positive lesions are related to prognosis; there is no direct linear relationship between the level of FES uptake and clinical outcomes. The FES/FDG ratio may reflect the two biological functions of hormone receptors and glucose metabolism in metastases and may be more valuable for predicting fulvestrant treatment. Third, the sample size was relatively modest. Despite the small cohort, the results were statistically significant.

## Conclusions

Our data suggest that dual ^18^F-FDG and ^18^F-FES PET imaging could be a potential predictor of efficacy to fulvestrant therapy among HR+HER2- MBC patients. These findings indicate that endocrine therapy should be individualized for patients with ER-positive MBC, particularly the presence of ^18^F-FES negative lesions.

## Data Availability Statement

The original contributions presented in the study are included in the article/**Supplementary Material**. Further inquiries can be directed to the corresponding authors.

## Ethics Statement

The studies involving human participants were reviewed and approved by Fudan University Shanghai Cancer Center Ethic Committee and Institutional Review Boards. Written informed consent for participation was not required for this study in accordance with the national legislation and the institutional requirements.

## Author Contributions

Conception and design: CL, ZY. Acquiring data, or analyzing and interpreting data: CL, XX, HY, YPZ. Drafting the manuscript: CL. Critically contributing to or revising the manuscript: SS, ZY. Enhancing its intellectual: ZY, SS, YJZ. All authors contributed to the article and approved the submitted version.

## Funding

This research is sponsored by Shanghai Sailing Program (20YF1408500), the Shanghai Committee of Science and Technology Fund (No.19ZR1411300), the Shanghai Engineering Research Center of Molecular Imaging Probes Program (No. 19DZ2282200) and Shanghai Municipal Health Commission (No.202040267).

## Conflict of Interest

The authors declare that the research was conducted in the absence of any commercial or financial relationships that could be construed as a potential conflict of interest.
