# Supplementary material for: Dual Tracers of 16α-[18F]fluoro-17β-Estradiol and [18F]fluorodeoxyglucose for Prediction of Progression-Free Survival After Fulvestrant Therapy in Patients With HR+/HER2- Metastatic Breast Cancer
Source: Front Oncol. 2020 Oct 29;10:580277. doi: 10.3389/fonc.2020.580277 (PMC7673439; doi:10.3389/fonc.2020.580277)
Supplement: Supplementary file 1 [file DataSheet_1.docx]

Based on the median value of FDG SUVmax was 4.4, 17patients with low FDG uptake (FDG SUVmax <4.4) and 18 FDG avid. Patients with high FDG uptake further divide into two groups by the median FES (FES SUVmax 4.5): high FDG/high FES (FES SUVmax ≥ 4.5, n= 11) and high FDG/low FES (FES < 4.5, n = 7). The Kaplan-Meier plots indicated that patients with low FDG uptake had a median PFS of 15.5 months. Of patients with more FDG avid tumors, with high average FES uptake had a median PFS of 7.7months and low FES uptake had a median PFS of 5.6. It is regrettable that the groups did not help to predict the prognosis (P = 0.234, Supplement Figure 1).


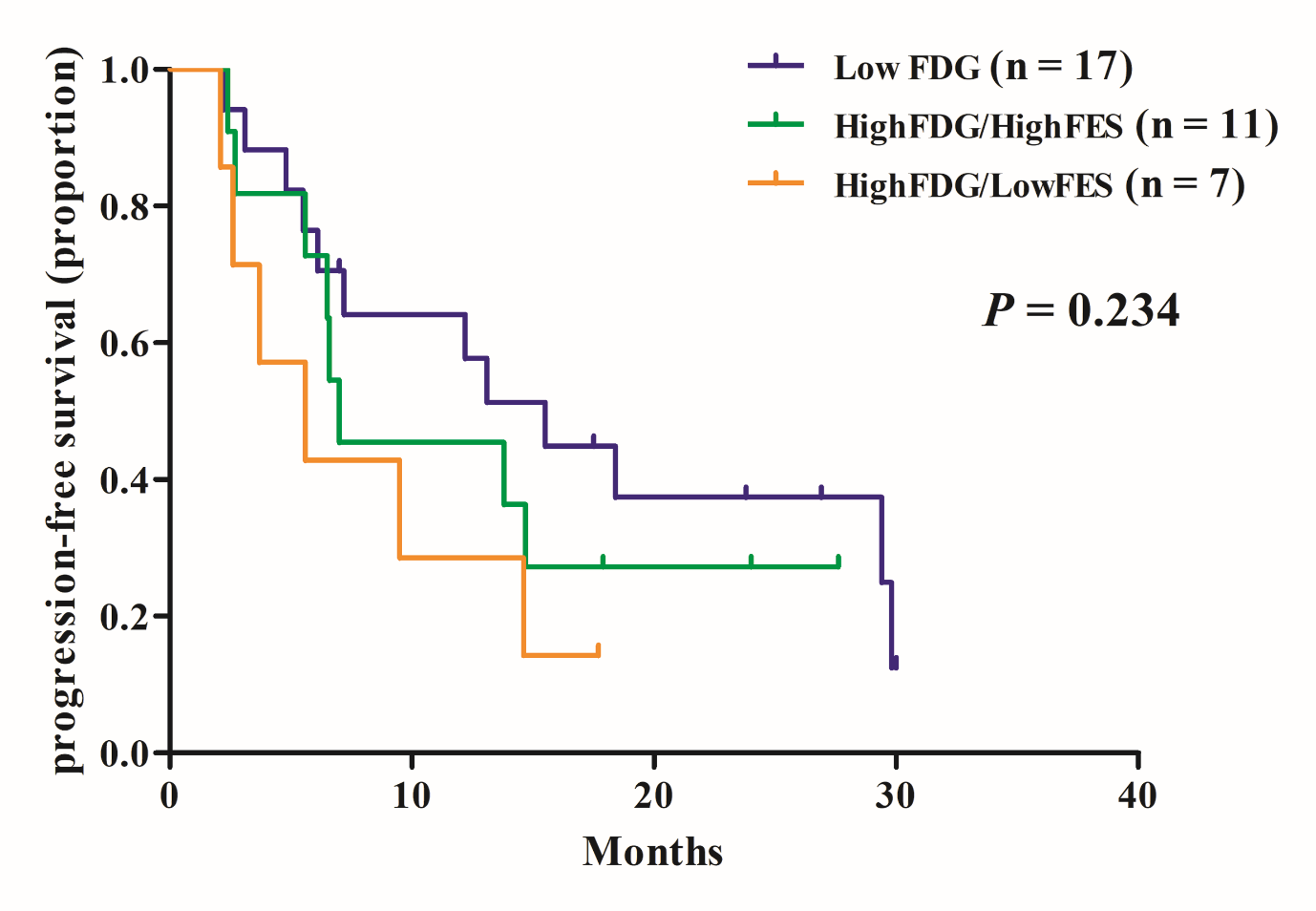


Figure 1. Kaplan-Meier estimates of the progression-free survival for patients stratified by low FDG, high FDG/high FES and high FDG/low FES.
