# Supplementary material for: Dual Tracers of 16α-[18F]fluoro-17β-Estradiol and [18F]fluorodeoxyglucose for Prediction of Progression-Free Survival After Fulvestrant Therapy in Patients With HR+/HER2- Metastatic Breast Cancer
Source: Front Oncol. 2020 Oct 29;10:580277. doi: 10.3389/fonc.2020.580277 (PMC7673439; doi:10.3389/fonc.2020.580277)
Supplement: Supplementary file 2 [file Table_1.docx]

**Supplement Table 1. PET parameters and clinical outcomes of individual patients.**

| Patient | Tumor sites | No. of lesions  (n) | FES Heterogeneity | Median  FES-SUVmax | Median  FDG-SUVmax | Median  FES/FDG Ratio | PFS (month) |
| --- | --- | --- | --- | --- | --- | --- | --- |
| 1 | Bone* | 6 | Yes | 5.5 | 3.9 | / | 3.1 |
|  | Lymph node | 1 |  |  |  |  |  |
| 2 | Bone | 2 | No | 2.7 | 4.7 | 0.59 | 2.6 |
|  | Lymph node | 1 |  |  |  |  |  |
| 3 | Bone | 11 | No | 5.6 | 5.1 | 1.12 | 2.4 |
| 4 | Bone | 1 | No | 6.9 | 2.6 | 2.97 | 13.1 |
|  | Lymph node | 4 |  |  |  |  |  |
| 5 | Bone* | 2 | Yes | 3.8 | 2.7 | / | 2.2 |
| 6 | Pleura | 3 | No | 2.7 | 3.7 | 0.74 | 30.0+ |
| 7 | Lymph node | 4 | No | 4.1 | 3.4 | 1.05 | 7.2 |
| 8 | Bone* | 8 | Yes | 6.0 | 6.9 | / | 7.0 |
|  | Lymph node | 1 |  |  |  |  |  |
| 9 | Breast | 1 | No | 2.5 | 10.6 | 0.21 | 14.6 |
|  | Bone | 1 |  |  |  |  |  |
|  | Lymph node | 2 |  |  |  |  |  |
| 10 | Bone | 7 | No | 4.2 | 3.8 | 1.09 | 15.5 |
|  | Lymph node | 2 |  |  |  |  |  |
|  | Lung | 1 |  |  |  |  |  |
| 11 | Bone* | 7 | Yes | 2.7 | 5.7 | / | 2.1 |
| 12 | Lymph node | 1 | No | 3.3 | 3.3 | 0.99 | 7.0+ |
| 13 | Bone | 11 | No | 3.4 | 6.5 | 0.78 | 17.7+ |
| 14 | Lymph node | 7 | No | 10.9 | 4.8 | 2.32 | 14.7 |
| 15 | Breast | 1 | Yes | 3.0 | 11.8 | / | 9.5 |
|  | Lymph node* | 8 |  |  |  |  |  |
| 16 | Lymph node | 2 | No | 3.9 | 4.1 | 0.90 | 17.5+ |
|  | Lung | 7 |  |  |  |  |  |
| 17 | Breast | 2 | No | 7.3 | 4.9 | 0.71 | 6.6 |
|  | Bone | 3 |  |  |  |  |  |
|  | Lymph node | 4 |  |  |  |  |  |
| 18 | Soft tissue | 1 | Yes | 2.0 | 4.2 | / | 5.5 |
|  | Lymph node* | 1 |  |  |  |  |  |
| 19 | Bone | 4 | Yes | 4.5 | 6.1 | / | 13.8 |
|  | Lymph node* | 14 |  |  |  |  |  |
|  | Lung | 2 |  |  |  |  |  |
| 20 | Breast | 1 | No | 6.4 | 15.5 | 0.52 | 27.6+ |
|  | Bone | 3 |  |  |  |  |  |
|  | Lymph node | 3 |  |  |  |  |  |
| 21 | Bone | 6 | Yes | 9.0 | 4.7 | / | 2.7 |
|  | Lymph node | 2 |  |  |  |  |  |
|  | Pleura | 2 |  |  |  |  |  |
|  | Lung* | 2 |  |  |  |  |  |
|  | Liver | 1 |  |  |  |  |  |
| 22 | Soft tissue | 1 | No | 5.8 | 4.3 | 1.30 | 26.9+ |
|  | Bone | 1 |  |  |  |  |  |
| 23 | Breast | 1 | No | 3.3 | 3.4 | 0.96 | 18.4 |
|  | Bone | 1 |  |  |  |  |  |
| 24 | Bone | 2 | No | 12.5 | 6.6 | 2.06 | 5.6 |
|  | Lymph node | 4 |  |  |  |  |  |
|  | Lung | 1 |  |  |  |  |  |
| 25 | Lymph node | 1 | No | 2.9 | 4.1 | 0.71 | 29.8 |
| 26 | Lymph node* | 3 | Yes | 6.2 | 3.2 | / | 6.1 |
| 27 | Lymph node | 1 | No | 2.5 | 3.8 | 0.66 | 12.2 |
| 28 | Pleura | 1 | No | 9.4 | 3.9 | 0.87 | 29.4 |
|  | Lymph node | 2 |  |  |  |  |  |
| 29 | Breast | 1 | No | 4.9 | 7.2 | 0.73 | 24.0+ |
|  | Lymph node | 5 |  |  |  |  |  |
| 30 | Breast | 1 | No | 6.3 | 5.2 | 1.10 | 17.9+ |
|  | Lymph node | 1 |  |  |  |  |  |
|  | Lung | 1 |  |  |  |  |  |
| 31 | Breast* | 1 | Yes | 2.7 | 4.4 | / | 5.6 |
|  | Bone | 17 |  |  |  |  |  |
|  | Lymph node* | 1 |  |  |  |  |  |
| 32 | Soft tissue | 2 | Yes | 3.0 | 6.1 | / | 3.7 |
|  | Bone* | 5 |  |  |  |  |  |
|  | Lymph node | 2 |  |  |  |  |  |
|  | Pleura | 1 |  |  |  |  |  |
| 33 | Bone | 4 | No | 7.0 | 2.1 | 2.78 | 4.8 |
|  | Lymph node | 1 |  |  |  |  |  |
| 34 | Bone | 15 | No | 13.5 | 4.3 | 3.23 | 23.8+ |
| 35 | Soft tissue | 1 | Yes | 5.8 | 5.5 | / | 6.5 |
|  | Lymph node | 1 |  |  |  |  |  |
|  | Pleura | 2 |  |  |  |  |  |
|  | Lung* | 1 |  |  |  |  |  |

**Abbreviations:** +, ongoing; *, with FES negative lesion(s).
